# Supplementary material for: Delivery of Alpha-Mangostin Using Cyclodextrins through a Biological Membrane: Molecular Dynamics Simulation
Source: Molecules. 2020 May 29;25(11):2532. doi: 10.3390/molecules25112532 (PMC7321106; doi:10.3390/molecules25112532)
Supplement: Supplementary file 1 [file molecules-25-02532-s001.pdf]

molecules

MDPI

Supporting Information for

Delivery of alpha-mangostin using cyclodextrin through a biological membrane: Molecular dynamics simulation

Wiparat Hotarat [1], Bodee Nutho [1], Peter Wolschann [2,3], Thanyada Rungrotmongkol [4,5,6*] and Supot Hannongbua [1,*]

[1] Center of Excellence in Computational Chemistry (CECC), Department of Chemistry, Faculty of Science, Chulalongkorn University, Bangkok 10330, Thailand
[2] Department of Pharmaceutical Chemistry, University of Vienna, Vienna 1090, Austria
[3] Institute of Theoretical Chemistry, University of Vienna, Vienna 1090, Austria
[4] Program in Bioinformatics and Computational Biology, Faculty of Science, Chulalongkorn University, Bangkok 10330, Thailand
[5] Structural and Computational Biology Research Unit, Department of Biochemistry, Faculty of Science, Chulalongkorn University, Bangkok 10330, Thailand
[6] Molecular Sensory Science Center, Faculty of Science, Chulalongkorn University, Bangkok 10330, Thailand

* E-mail: t.rungrotmongkol@gmail.com, thanyada.r@chula.ac.th; Fax: +66(0)2218-5418;
* E-mail: supot.h@chula.ac.th.com; Fax: +66(0)2218-7603

Keywords: Alpha-mangostin; cellular membrane; POPC; cyclodextrins; drug penetration, molecular dynamics, PMF

The pysical properties and molecular dimensions of CDs

Table S1. The physical properties and molecular dimensions of α-, β-, and γCDs[1].

Type of CDs	No. of Glucose units	Molecular weight (g/mol)	Molecular diameter (nm)			Cavity volume (nm)
			Inside	Outside	Height	
α	6	973	0.57	1.37	0.7	17.5
β	7	1153	0.78	1.53	0.7	26.1
γ	8	1297	0.95	1.69	0.7	42.8

Force field parameters

The initial structure of 128 POPC membrane (64 POPC per leaflet) was constructed using the CHAMM-GUI membrane builder [2-4]. The obtained lipid membrane was solvated with 12,800 TIP3P water molecules in a simulation box of 102.77 × 103.96 × 91.89 $Å^3$. The topology and coordinate in AMBER format were prepared using the tleap module implemented in the AMBERTOOL16 software. The AMBER Lipid14 force field was used to describe the POPC membrane [5], whereas the MGS parameters were obtained from a previous study [6]. To treat the βCD and DMβCD molecules, the GLYCAM06 force field [7] was applied.

Permeability of MGS

To investigate the permeation of MGS into the POPC membrane, the distance along z-direction of each ring of MGS (A-ring or C-ring) to the center of the lipid bilayer (z = 0) was plotted in **Fig. S1**. The results showed that the A-ring firstly entered into the hydrophobic region of the lipid membrane,

while the C-ring pointed towards the water layer of the lipid membrane. In the first 100 ns, the MGS was rapidly dipped into the membrane surface. After that, the MGS was located and reached an equilibrium below the lipid head groups at 0.94 ± 0.18 nm from the center of the lipid bilayer. The MGS perpendicularly interacted with the POPC surface and was then oriented parallel to the phosphate groups of the lipid bilayer. In **Fig. S1(d)-(f)**, the initial conformation was started by placing the C-ring of MGS (C-MGS) in a location close to the lipid surface. Along the simulation, the MGS rotated its conformation at the outer surface of the membrane by pointing the A-ring towards the lipid surface, and then MGS penetrated deeper into the inner membrane of the lipid bilayer. After that, MGS reached an equilibrium underneath the polar head groups at 1.06 ± 0.16 nm from the center of the lipid bilayer. From these MD trajectories, we imply that MGS always turned its A-ring to face towards the polar head groups of the membrane. Both the A-MGS and C-MGS forms vertically interacted with the phosphate groups. However, prolonged simulations over 100 ns indicated that MGS was horizontal to the lipid alkyl groups. In all cases, the MGS had no ability to penetrate across the other side of lipid bilayer in both initial forms of the 500-ns MD simulations. Noticeably, the isoprenyl groups of the A-ring enhanced the hydrophobic properties of MGS, and so the MGS could easily penetrate into the inner membrane of the lipid bilayer. These results are in good agreement with a previous report on the penetration of MGS into a bacterial membrane consisting of POPE and POPG at a 75:25 ratio [8]. Their results revealed that the presence of the isoprenyl groups increased the hydrophobicity of MGS and led to an enhanced drug penetration through the lipid bilayer.

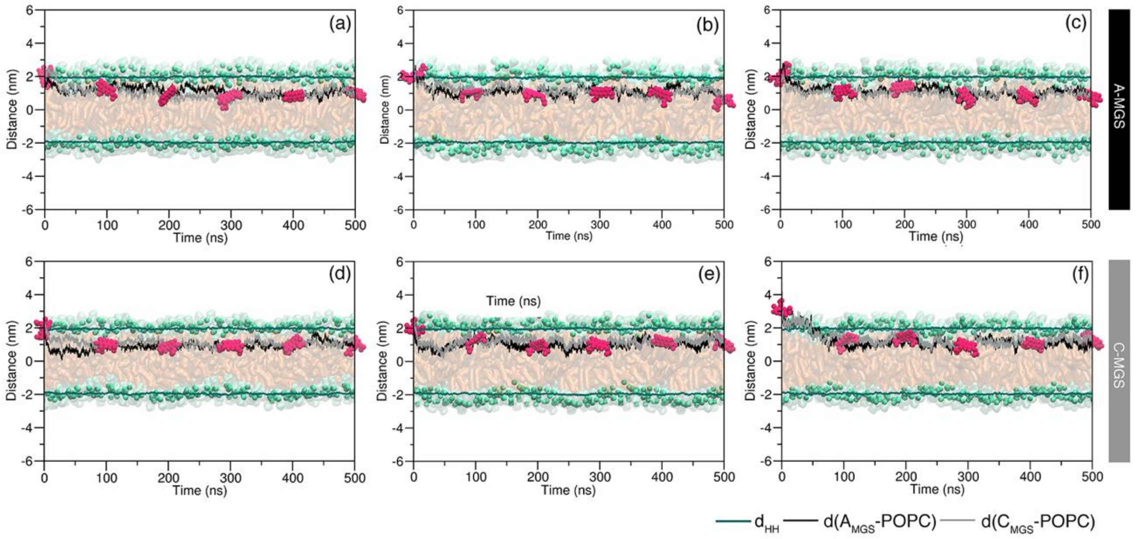

Figure S1. Penetration of MGS into the interior membrane of lipid bilayer for (**a**)-(**c**) A-MGS, and (**d**)-(**f**) C-MGS. The distance between the two polar head groups, the distance between the COM of the A-ring of MGS and the COM of the POPC (z = 0 nm), as well as the distance between the COM of the C-ring of MGS and the COM of the POPC are represented by a green line (d_{HH}), black dot [$d(A_{MGS}$-POPC)], and grey dot [$d(C_{MGS}$-POPC)], respectively.

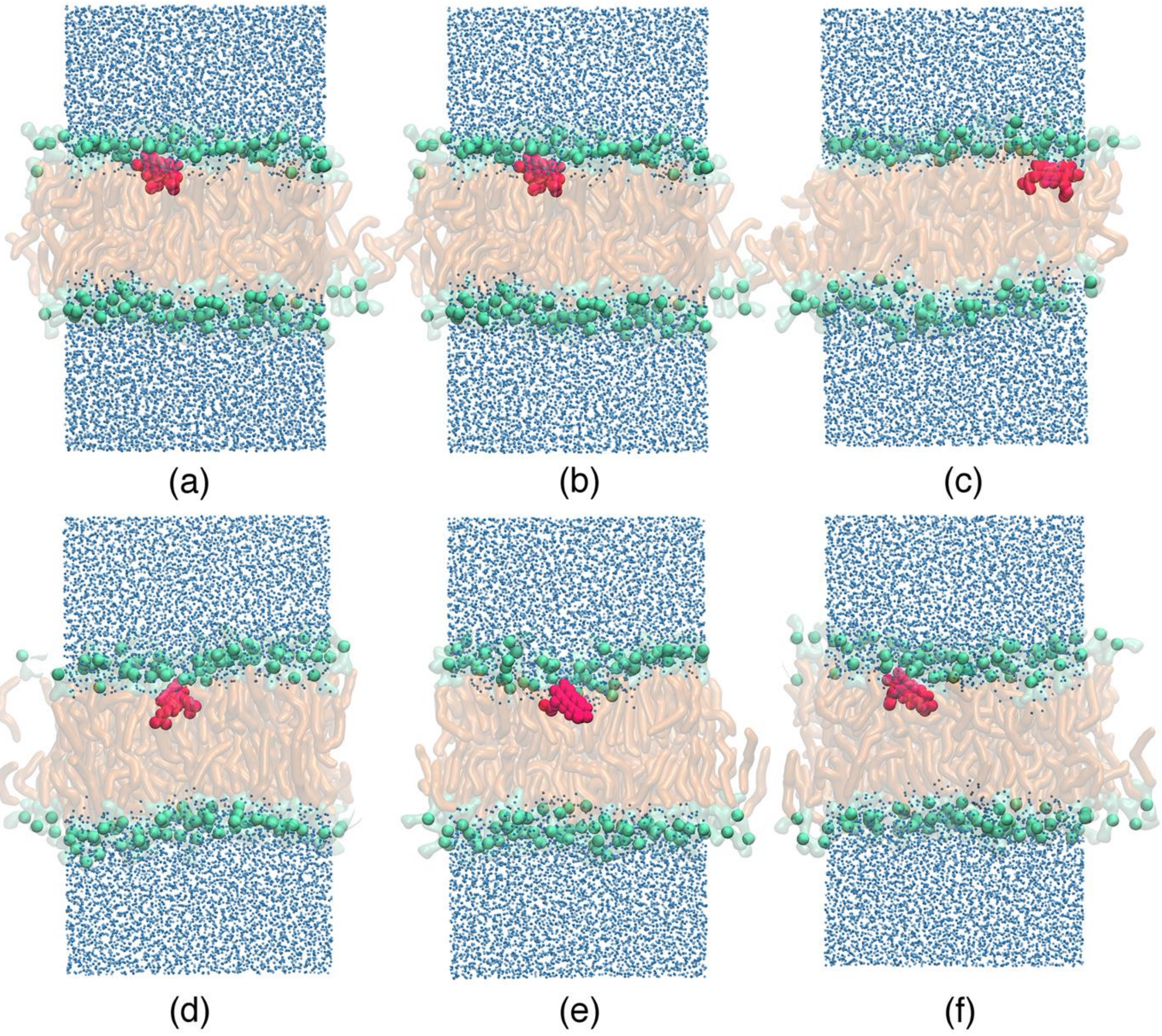

Figure S2. The last MD snapshots for the MGS adsorption on the lipid bilayer for (**a**)-(**c**) A-MGS, and (**d**)-(**f**) C-MGS, respectively.

To determine the drug interaction with the lipid bilayer, the degree of H-bonds was observed using the default geometric criteria of GROMACS, using distance between the hydrogen donor (D) and acceptor (A) of ≤ 0.35 nm and a D···H–D angle of ≤ 30°. Plots of the number of H-bonds *versus* simulation time for A-MGS and C-MGS are presented in **Fig. S3**. The two different starting conformations (A-MGS and/or C-MGS) preferentially formed H-bonds with the phosphate groups of the lipid membrane rather than the glycerol ester groups. Interestingly, the number of H-bonds between MGS and the polar head groups of the lipid bilayer corresponded to the favorable location on the lipid membrane. We observed a higher number of H-bonds between MGS and the phosphate groups of the POPC membrane in the first 200 ns for A-MGS [**Fig. S3**(a)], which then decreased from 3 to 1 in the last 200 ns. For C-MGS, shown in **Fig. S3**(b), the MGS frequently formed H-bonds with the phosphate groups rather than with the glycerol ester groups of the lipid membrane, as in the case of A-MGS, corresponding to its equilibrated position on the membrane.

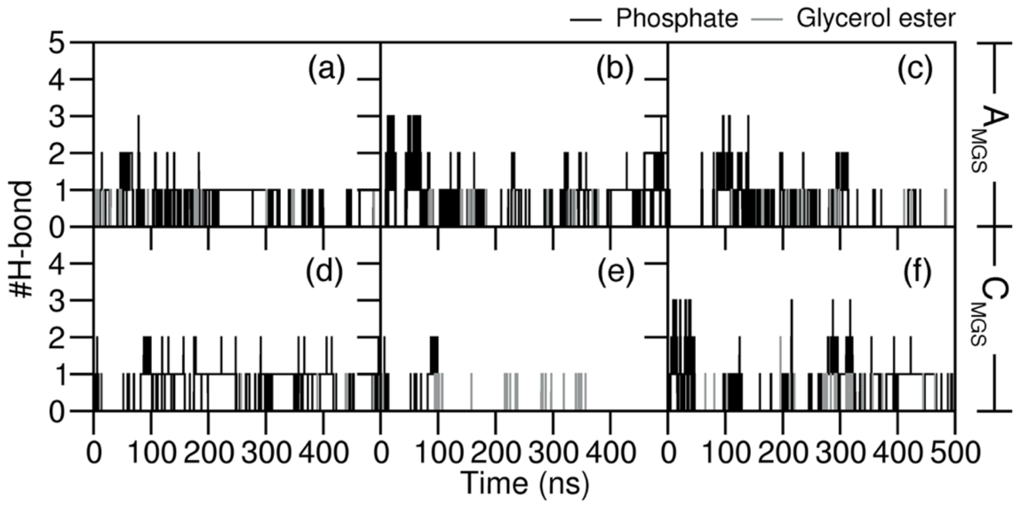

Figure S3. Time evolution of the number of H-bonds of (**a**) A-MGS and (**c**) C-MGS with the polar head groups of the lipid bilayer (phosphate and glycerol esters). The H-bond interactions of MGS with the phosphate and glycerol ester groups are represented by a black line and light grey line, respectively.

Permeability of A-MGS/CDs and C-MGS/CDs inclusion complexes

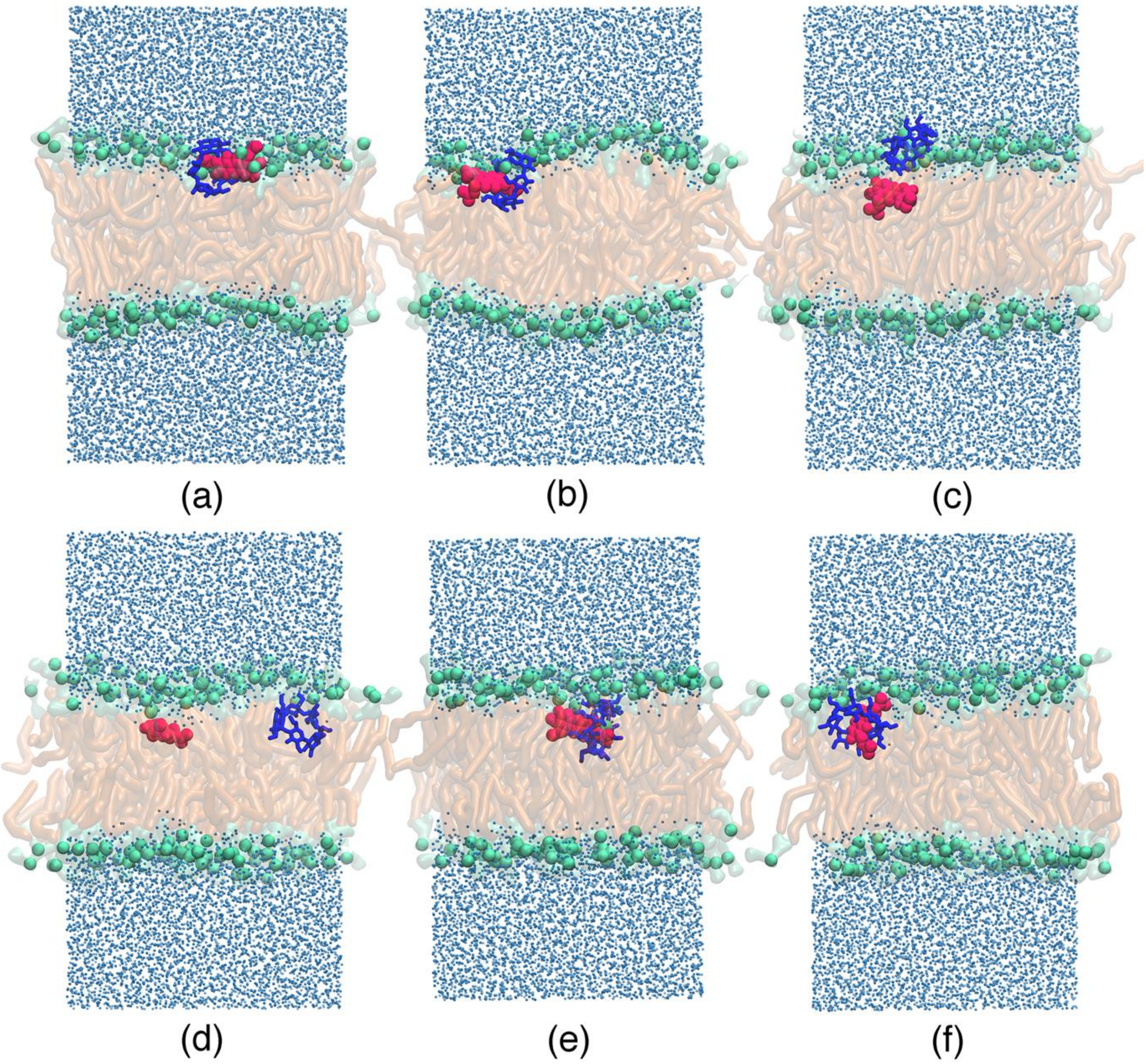

Figure S4. The last MD snapshots for the inclusion complexes adsorption on the lipid bilayer for (**a**)-(**c**) A-MGS/βCD, and (**d**)-(**f**) A-MGS/DMβCD, respectively. The MGS and CDs are represented by a red ball and blue stick model, respectively.

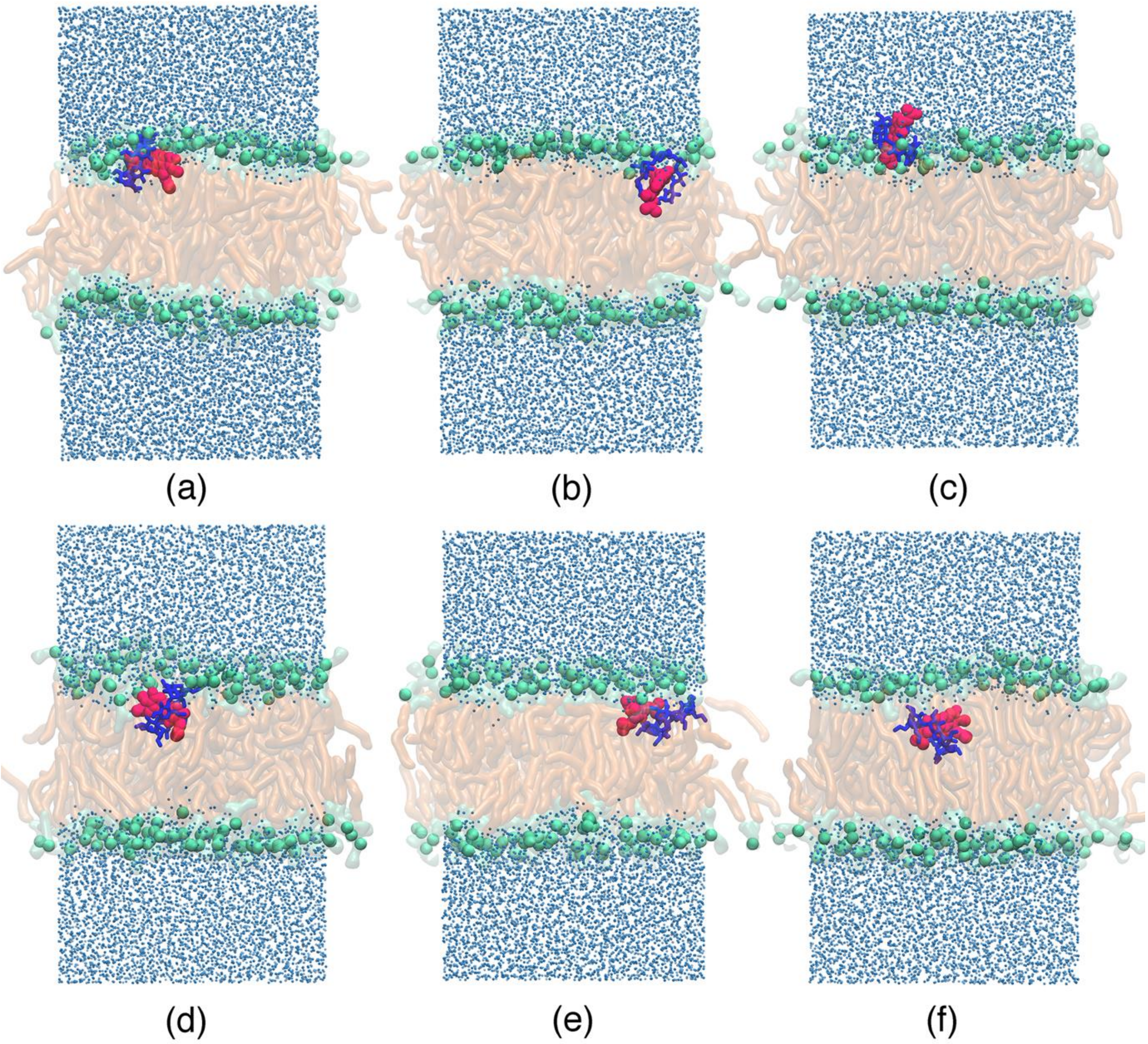

Figure S5. The last MD snapshots for the inclusion complexes adsorption on the lipid bilayer for (**a**)-(**c**) C-MGS/βCD, and (**d**)-(**f**) C-MGS/DMβCD, respectively. The MGS and CDs are represented by a red ball and blue stick model, respectively.

The free energy profile

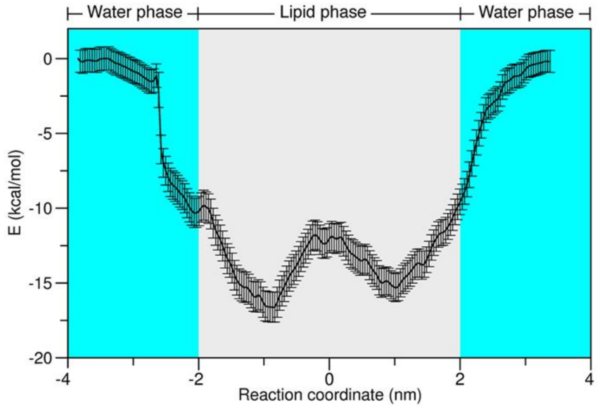

Figure S6. The free energy profile for MGS penetration through the POPC membrane.

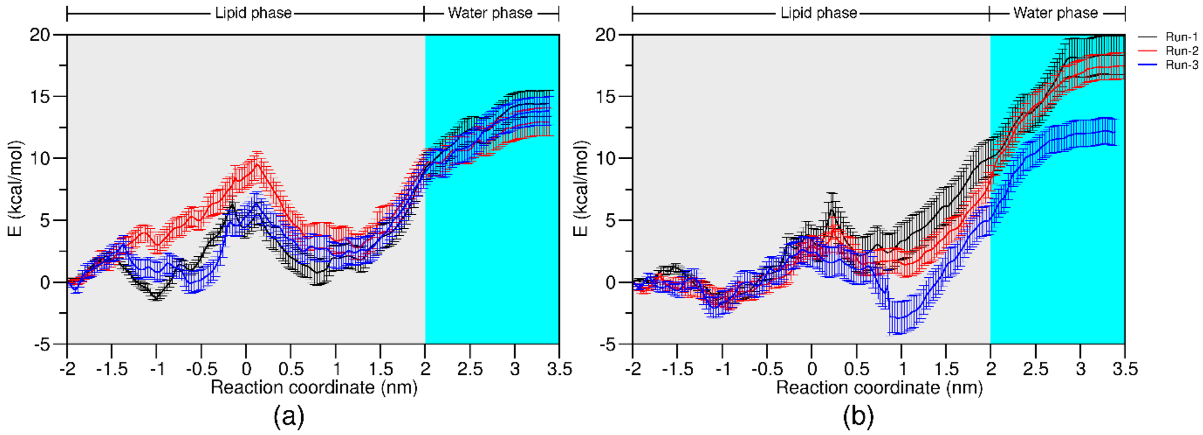

Figure S7. Triplicate free energy profiles for (a) A-MGS/βCD and (b) MGS/DMβCD.

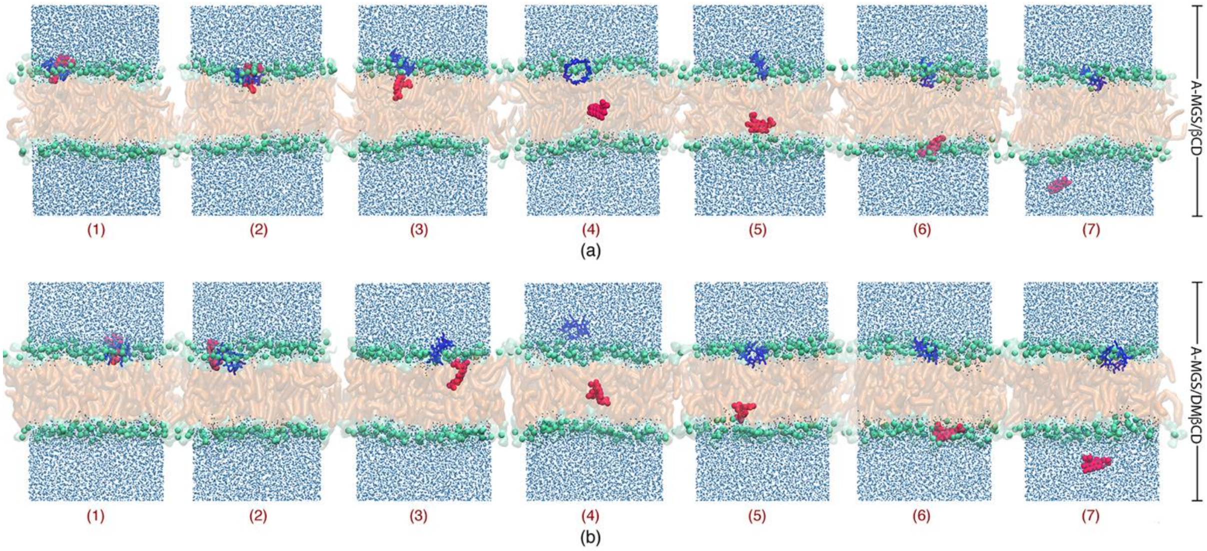

Figure S8. Representation (1) – (7) of each umbrella sampling point of (a) A-MGS/βCD and (b) A-MGS/DMβCD.

Prolonged simulation

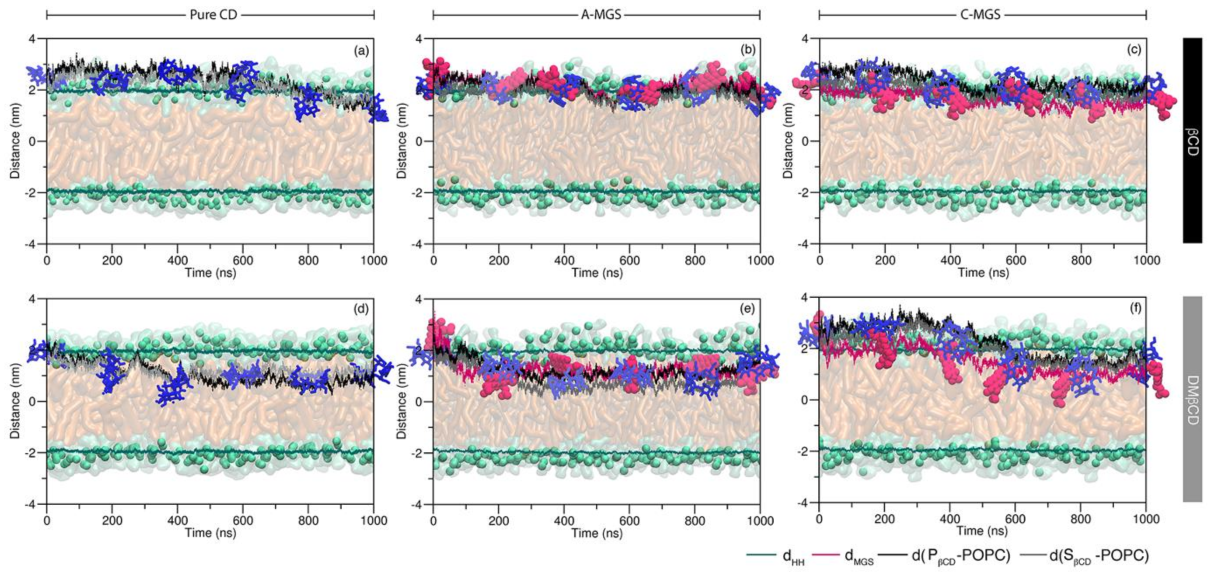

Figure S9 Prolonged simulation (1 μs) of (a) βCD, (b) A-MGS, (c) C-MGS, (d) DMβCD, (e) A-MGS/βCD, and (f) C-MGS/DMβCD.

References

1. Szejtli, J., Introduction and General Overview of Cyclodextrin Chemistry. *Chemical Reviews* **1998,** *98* (5), 1743-1754.

2. Jo, S.; Lim, J. B.; Klauda, J. B.; Im, W., CHARMM-GUI Membrane Builder for mixed bilayers and its application to yeast membranes. *Biophys J* **2009,** *97* (1), 50-8.

3. Jo, S.; Kim, T.; Im, W., Automated builder and database of protein/membrane complexes for molecular dynamics simulations. *PLoS One* **2007,** *2* (9), e880.

4. Qi, Y.; Cheng, X.; Lee, J.; Vermaas, J. V.; Pogorelov, T. V.; Tajkhorshid, E.; Park, S.; Klauda, J. B.; Im, W., CHARMM-GUI HMMM Builder for Membrane Simulations with the Highly Mobile Membrane-Mimetic Model. *Biophys J* **2015,** *109* (10), 2012-22.

5. Dickson, C. J.; Madej, B. D.; Skjevik, A. A.; Betz, R. M.; Teigen, K.; Gould, I. R.; Walker, R. C., Lipid14: The Amber Lipid Force Field. *J Chem Theory Comput* **2014,** *10* (2), 865-879.

6. Rungnim, C.; Phunpee, S.; Kunaseth, M.; Namuangruk, S.; Rungsardthong, K.; Rungrotmongkol, T.; Ruktanonchai, U., Co-solvation effect on the binding mode of the alpha-mangostin/beta-cyclodextrin inclusion complex. *Beilstein J Org Chem* **2015,** *11*, 2306-2317.

7. Kirschner, K. N.; Yongye, A. B.; Tschampel, S. M.; Gonzalez-Outeirino, J.; Daniels, C. R.; Foley, B. L.; Woods, R. J., GLYCAM06: a generalizable biomolecular force field. Carbohydrates. *J Comput Chem* **2008,** *29* (4), 622-55.

8. Koh, J. J.; Qiu, S.; Zou, H.; Lakshminarayanan, R.; Li, J.; Zhou, X.; Tang, C.; Saraswathi, P.; Verma, C.; Tan, D. T.; Tan, A. L.; Liu, S.; Beuerman, R. W., Rapid bactericidal action of alpha-mangostin against MRSA as an outcome of membrane targeting. *Biochim Biophys Acta* **2013,** *1828* (2), 834-44.
